# Sensitive Identification of Microcystin-LR via a Reagent-Free and Reusable Electrochemical Biosensor Using a Methylene Blue-Labeled Aptamer

**DOI:** 10.3390/bios12080556

**Published:** 2022-07-22

**Authors:** Xiaoqian Wei, Shanlin Wang, Yujuan Zhan, Tianhan Kai, Ping Ding

**Affiliations:** 1 Xiang Ya School of Public Health, Central South University, Changsha 410078, China; xiaoqianwei@csu.edu.cn (X.W.); 18273149872@163.com (S.W.); 216911040@csu.edu.cn (Y.Z.); 2 Hunan Provincial Key Laboratory of Clinical Epidemiology, Changsha 410078, China

**Keywords:** microcystin-LR, methylene blue, aptamer, electrochemical sensor

## Abstract

We report a methylene blue (MB)-modified electrochemical aptamer (E-AB) sensor for determining microcystin-LR (MC-LR). The signal transduction of the sensor was based on changes in conformation and position of MB induced by the binding between MC-LR and the modified aptamer probe. In the absence of MC-LR, an aptamer probe was considered partially folded. After combining aptamer and MC-LR, the configuration of the aptamer probe changed and facilitated the electron transfer between MB and the electrode surface. As a result, an increased current response was observed. We optimized the parameters and evaluated the electrochemical performance of the sensor using square wave voltammetry (SWV). MC-LR was measured from 1.0 to 750.0 ng/L with a detection limit of 0.53 ng/L. The reliability of the method was verified by the determination of MC-LR in environmental real samples, such as pond water and tap water. Moreover, we demonstrated that this reagent-less biosensor could be regenerated and reused after rinsing with deionized water with good accuracy and reproducibility. As a reusable and regenerable E-AB sensor, this rapid, reagent-free, and sensitive sensing platform will facilitate routine monitoring of MC-LR in actual samples.

## 1. Introduction

Cyanobacteria are organisms whose origins date back billions of years and are ubiquitous in all aquatic environments. The eutrophication of lakes and reservoirs leads to the proliferation of harmful cyanobacteria worldwide, which poses an increasingly serious threat to public health. Microcystins (MCs), believed to be linked to algal blooms, are among the most frequently detected cyanogen toxins in freshwater globally and pose a major threat to the quality of drinking water and ecosystems. MCs are cyclic heptapeptides consisting of two variable positions and five constant amino acids [1]. Due to its circular heptapeptide structure, it has a strong affinity and high specificity for serine/threonine protein phosphatase and therefore acts as an effective inhibitor of the eukaryotic protein phosphatase family PP1 and PP2A, leading to cell apoptosis. In addition, MCs can cause oxidative stress in the liver and may lead to death. Differences in toxicity between microcystin strains are attributed to the content of the peptide synthase produced by microcystin [2,3]. Microcystin-LR is considered the most toxic form of MCs, with an LD_50_ of 43 µg/kg [3,4]. In 1998, the guideline value of MC-LR for drinking water, established by the WHO, was 1 µg/L [5].

Many tools were utilized to identify and quantify MCs and related toxins, such as HPLC-MS and ELISA. HPLC-MS is an analytical method approved by EPA in the United States to detect various algal toxins with detection limits ranging from 0.1 to 1 g/L [6,7]. However, chromatography requires extensive sample pretreatment compared with immunoassays, which are sensitive to trace samples and are easy to use in routine screening of actual samples [8]. It is difficult to distinguish the structurally similar microcystin homologs, especially in the presence of other highly abundant but less toxic homologs [9]. Therefore, there is an urgent need to develop an advanced, small and portable device that can overcome the shortcomings of current methods for rapid and sensitive measurement of biological compounds [10,11].

Electrochemical sensors are suitable for in situ monitoring because of their potential miniaturization, portability, and automation for biosensor applications [12]. In addition, electrochemical sensors have the advantages such as high specificity, low detection limit, and low cost [8,10,13]. Progress was made in developing specific and highly sensitive electrochemical biosensors/immunosensors for environmental monitoring, particularly for monitoring MC-LR [1,14,15]. Electrochemical biosensors developed for detecting phycotoxins usually have three main components: a biometric element, a signal transduction mechanism, and a sensor electrode. Biometric receptors are important for the selectivity and specificity of electrochemical biosensors. Antibodies allow for a highly specific molecular recognition, which is the most used receptors in the electrochemical biosensing of MC-LR [16,17,18]. However, the detection by these electrochemical sensors depends on conformational changes caused by antigen/antibody binding and thus, is suitable for detecting large analytes. Detecting algal toxins (<1 kDa) using an electrochemical biosensor often fails to achieve desired results. Aptamers used as biometrics for the electrochemical detection of algal toxins are a combination of small single-stranded DNA/RNA molecules with higher stability than antibodies when immobilized on the electrode surface [19,20,21] and thus, have great potential for development of commercial sensors [4,22,23]. The aptamer terminus typically modifies disulfide groups, amino groups, ferrocene, and thiols, which can readily bind to electrode surfaces [24,25]. The electrochemical aptamer-based (E-AB) sensor is a special biosensor that uses stationary aptamers with Redox tags. In these sensors, the interaction between the target and aptamer induces the current change as the distance between MB and electrode surface and the electron transfer (ET) efficiency from the tag to the electrode surface are altered [26]. A series of electroactive Redox probes, such as [Fe(CN)_6_]^3−/4−^ and MB, were used to facilitate the voltammetric detection of algal toxins [27]. MB is a well-studied and commonly used Redox label with high stability [28]. The change in aptamer conformation induced by target-aptamer binding results in the alteration of ET efficiency [29,30,31]. To fulfill the unmet need for rapid analysis of MC-LR, we designed and fabricated a novel, simple, and rapid MB-modified E-AB sensor that is capable of analyzing MC-LR. Specifically, we report for the first time the use of an MB-modified aptamer as a biometric receptor in the electrochemical sensor to detect MC-LR. We systematically evaluated the sensor’s response using square wave voltammetry (SWV) and cyclic voltammetry (CV). Our results demonstrate that the E-AB sensor can detect MC-LR with high specificity. This work provides insights into the design of displacement-based biosensors and guides preparations of surface-immobilized E-AB sensors for MC-LR determination in the future.

## 2. Experimental Section

### 2.1. Apparatus and Reagents

The electrochemical measurements were conducted using a CHI660E electrochemical analyzer (Chenhua Instruments, Shanghai, China). An Au disc-electrode (2 mm in diameter, CHI101, Chenhua Instruments, Shanghai, China), an Ag/AgCl electrode (CHI111, Chenhua Instruments, Shanghai, China), and a platinum wire (CHI115, Chenhua Instruments, Shanghai, China) were used as the working electrode, reference electrode and counter electrode, respectively. Standard samples of Microcystin-YR, Microcystin-LR, and Microcystin-RR (purity ≥ 95%) were acquired from Algalchem Inc. (Taiwan, China). Aluminum oxide powder (Al_2_O_3_, 1, 0.3, 0.05 μm), ethanol, hydrogen peroxide (H_2_O_2_, 30%), magnesium chloride (MgCl_2_, 99%), sodium chloride (NaCl, 99%), potassium chloride (KCl, 99%), sulfuric acid (H_2_SO_4_), potassium nitrate (KNO_3_, 99%) and tris-(2-carboxyethyl) phosphine hydrochloride (TCEP, 99%) were acquired from China Pharmaceutical Group Chemical Reagent Co. Ltd. (Shanghai, China). The 6-mercapto-1-hexanethiol (MCH) was obtained from Aladdin (Shanghai, China). Aptamers, standard samples, TCEP, and MCH, were prepared by dissolving the toxins in a Tris-HCl buffered solution (20 mM Tris, pH 7.5, 5 mM MgCl_2_, 10 mM NaCl). All solutions were prepared using ultrapure water with a resistance of 18.2 MΩ·cm^−^^1^. The thiolated and MB-modified aptamer for MC-LR [22] was synthesized by Shanghai Sangon Biotechnology Co., Ltd. (Shanghai, China).

### 2.2. Sensor Preparation

Before modification, the Au electrode was polished using Al_2_O_3_ with different particle sizes successively (1, 0.3, and 0.05 μm). The electrode was ultrasonicated for 5 min in ultrapure water and immersed in fresh piranha solution (mixed H_2_SO_4_ and 30% H_2_O_2_ in a ratio of 3:1) for 5 min. The electrode was rinsed with ethanol and ultrapure water for 5 min and was scanned in 0.1 M H_2_SO_4_ at the voltage between 0.0–1.6 V for 5 min to obtain the characteristic peaks. To obtain an aptamer-modified electrode, 10 mM TCEP was added into an aqueous solution containing 10 μM MC-LR aptamer. This solution was kept in the dark for 1 h to reduce disulfide bonds. Next, the electrodes were immersed in a solution containing 1 μM MC-LR aptamer for 12 h at room temperature and rinsed in a Tris-HCl buffered solution. After modification, the electrodes were incubated in a Tris-HCl buffered solution containing 1 mM MCH for 1 h, followed by a thorough washing before the measurements.

### 2.3. Electrochemical Measurements for MC-LR Determination

All electrochemical measurements were carried out at room temperature (25 °C). CV and SWV were performed in an aqueous solution containing 20 mM Tris-HCl using a three-electrode system. For MC-LR determination, the sensing platform was immersed into a Tris-HCl buffered solution containing MC-LR in different concentrations. SWV was scanned (0 to −0.5 V) after the electrode was incubated for 30 min in the same solution with the following parameters: frequency at 60 Hz, a step potential of 1 mV, and 25 mV amplitude. CV (0.1 to 0.6 V) was scanned with a scan rate of 0.1 V/s. The peak currents (ip) of MB were recorded. Each measurement was repeated at least three times to obtain the standard deviation.

## 3. Results and Discussion

### 3.1. E-AB Platform Design and Sensing Mechanism

**Figure 1:**
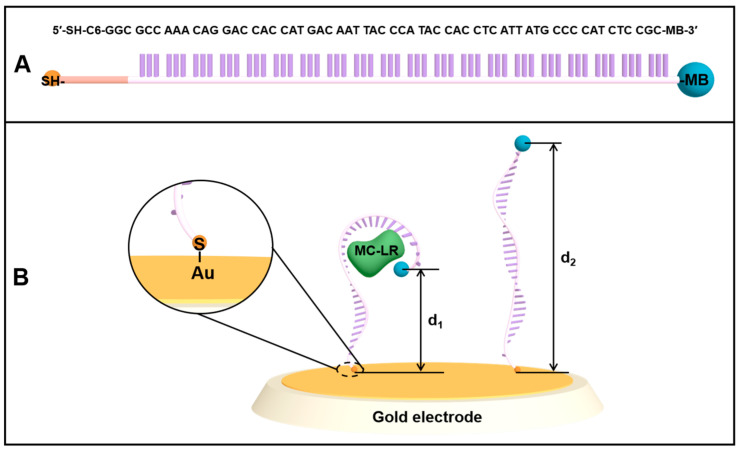
Schematic illustration of the fabrication steps of the sensor. (**A**) The thiolated and methylene blue (MB)−modified aptamer (5′−SH−C6-GGC GCC AAA CAG GAC CAC CAT GAC AAT TAC CCA TAC CAC CTC ATT ATG CCC CAT CTC CGC−MB−3′) for MC−LR; (**B**) The aptamer labeled with a methylene blue (MB) group at the 3′−terminus of the aptamer is immobilized on a gold electrode surface through the gold-sulfur chemistry. In the absence of target MC−LR, the MB tag at the 3′−terminus demonstrates low ET efficiency due to the possible interaction with aptamer bases or a relatively large distance from the electrode surface. The addition of MC−LR induces the conformational change of aptamer, which results in alterations in the local environment of MB, interactions between MB and aptamer bases, and the distance of MB to the electrode surface, bringing MB closer to the electrode surface and thus, enhancing ET efficiency.

**Figure 2:**
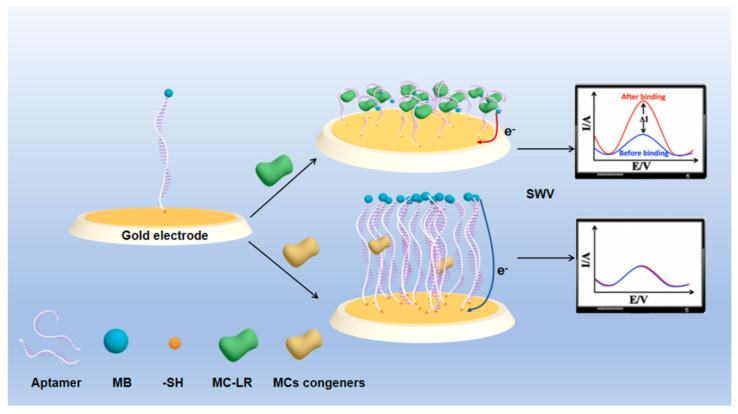
The working principle of the electrochemical sensor for MC−LR detection. In the absence of MC−LR, the methylene blue (MB)−modified aptamer probes are linear. However, in the presence of MC−LR, the probe is presumed to change its conformation, bringing MB closer to the electrode surface, thereby altering the ET kinetics between the MB label and the underlying gold electrode. This alteration in probe conformation and surface diffusivity of the MB label increases the MB current, which is exhibited as a “signal-on” sensor behavior.

The design of the (MB)-modified aptamer and E-AB sensor is illustrated in Figure 1. The 60 nt aptamer against MC-LR has a simple linear structure, which can specifically recognize and capture MC-LR (Figure 1A) [22]. The aptamer, with the mercaptan group introduced at the 5′-terminus, was modified on the electrode surface by combining the mercapto group with the gold surface. Meanwhile, the REDOX probe, MB, was modified at the 3′-terminus of the aptamer (Figure 1B), and the aptamer is partially folded without MC-LR. In addition, there is a wide ET distance between MB tags and the electrode surface (d_2_), and the electrochemical transfer rate is slow. After adding MC-LR, the aptamer interacts with MC-LR, resulting in the conformational changes of the aptamer, which leads to the proximity of MB to the electrode surface (d_1_). Such alterations facilitate the ET, thus increasing the electrochemical signal. The principle of the measurement of MC-LR using an E-AB sensor is well-described in Figure 2. The flexible surface attachment sites bend in the presence of MC-LR, which changes the ET rate, allowing the terminal redox reporter to strike the electrode surface [31]. In SWV analysis, the REDOX signal molecule, MB, produces a reduced current peak, and the increased value of MB current signal is measured for the quantitative determination of MC-LR.

### 3.2. Sensor Characterization Using CV and SWV

CV was utilized to analyze the response of the sensor to MC-LR under optimal conditions. Figure S1 displays the CV of the bare Au sensor in a 20 mM Tris-HCl buffered solution. A well-defined MB peak was observed at around −0.25 V (vs. Ag/AgCl, i.e., without MC-LR), verifying the successful modification of the aptamer (Figure S2A). MB current increased significantly when MC-LR was added, indicating that the optimum sensor responses to MC-LR (Figure S2B). The addition of MC-LR has led to the formation of folded aptamer, which is consistent with our proposed sensing mechanism.

### 3.3. Optimization of the Aptasensor Reaction Conditions

**Figure 3:**
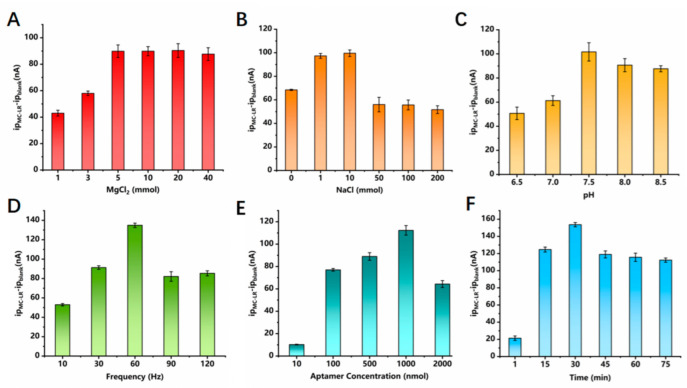
Optimization of the experimental conditions: (**A**) MgCl_2_ concentration; (**B**) NaCl concentration; (**C**) pH value; (**D**) frequency; (**E**) aptamer concentration; (**F**) modification time. All conditions were tested with 100 ng/L MC−LR.

To obtain the best analytical performance of the sensing platform, we optimized the reaction conditions, including the concentrations of Mg^2+^, Na^+^, and aptamer, pH, frequency, and modification time. Figure 3A reveals the influence of Mg^2+^ concentration on the current signal at the E-AB sensor in a 20 mM Tris-HCl buffer solution containing 100 ng/L MC-LR. The signal increased as the concentration of Mg^2+^ was increased and started to plateau when the Mg^2+^ concentration reached 5 mM. This is due to the enhanced interaction between the carboxyl or hydroxyl groups and MC-LR in the presence of Mg^2+^, which promotes the binding between MC-LR and the aptamer. Na^+^ concentration was also found to affect the electrochemical performance of the sensor. Figure 3B reveals that SWV currents increased with the increase in NaCl concentrations, indicating the role of salt ions in improving the conductivity of the solution and reducing the resistance to the solution. Additionally, salt ions can help to stabilize the secondary structure of the adapter chain. However, the current signal diminished when the NaCl was above 10 mM because the increased concentration of NaCl in the solution hampers the interaction between MC-LR and the aptamer. Figure 3C shows that the current response increased with increasing pH but remained constant when the pH reached 7.5, indicating that low pH can impact the conformation of DNA and MC-LR, which consequently diminishes the binding affinity of mercapto aptamer with MC-LR. In addition, acidic conditions affect the rate of ET signal to the electrode surface. The potential shifted toward a negative direction as the pH increased (Figure S3). The frequency of SWV was optimized to obtain the largest current responses. Figure 3D and Figure S4 show that the optimal SWV measurement frequency is 60 Hz. Furthermore, the effect of aptamer concentration was investigated, which revealed that increasing the aptamer concentration from 10 to 1000 nM significantly enhanced the peak current obtained at the E-AB sensor, but a further increase in the concentration resulted in diminished peak current (Figure 3E). The reaction kinetics between the aptamer and MC-LR (Figure 3F) was also investigated, which revealed that the current response reached the highest value after 30 min due to the saturation of MC-LR at the active sites. A slight decrease in the current was observed after 30 min, which was also reported in a previous study [27].

### 3.4. Sensor Sensitivity

**Figure 4:**
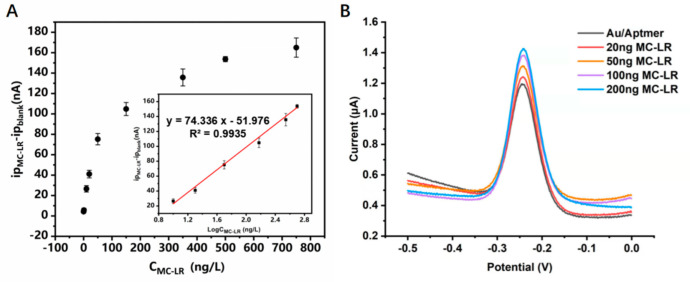
(**A**) Calibration curves for the electrochemical sensor obtained in SWV. The inset depicts the linear dynamic range, the slope (sensitivity), and the correlation coefficient (R^2^). (**B**) SWVs of the E−AB sensor in different concentrations of MC−LR solutions.

The LOD and sensitivity of the sensor were determined using SWV measurements since they offer better sensitivity and a distinct MB peak. We assessed the response of the sensor to different concentrations of MC-LR to obtain the LOD. The result indicates that the MB current increased with increasing MC-LR concentrations (Figure 4B). The linear dynamic range was between 1–750 ng/L, and the LOD was estimated to be 0.53 ng/L using the linear regression method. The *i*p value demonstrates linear relationships toward the logarithm of MC-LR concentration, which are expressed as y = 74.336x − 51.976 (R^2^ = 0.9935), where y represents ip_MC-LR_ − ip_blank_ (nA) and x represents LogC_MC-LR_ (ng/L) (Figure 4A). Table 1 compares the performance of the sensors proposed in this study with that of other electrochemical biosensors used for MC-LR detection. This sensor has a lower LOD than the majority of other electrochemical platforms.

### 3.5. Sensor Selectivity, Stability, and Reusability

**Figure 5:**
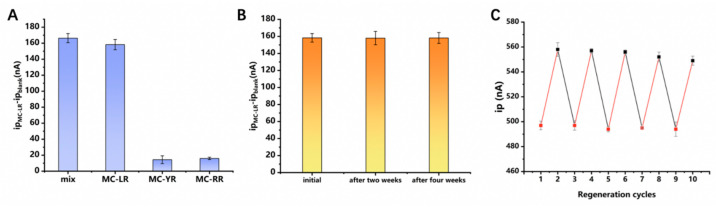
(**A**) Selectivity test of the aptamer sensor towards MC−LR through a comparison with the results obtained from using the mixture, MC−YR, and MC−YR, respectively; (**B**) Stability test of the aptamer sensor at initial, after two weeks, and after four weeks; the concentration of MC−LR in Figure 5A,B is 500 ng/L; (**C**) regeneration test of aptamer sensor for detection of MC−LR. The peak current, *i*_p_, was recorded in the absence/presence of 50 ng/L MC−LR after regeneration. The MC−LR−bound electrode was regenerated by rinsing with ultra−pure water for 30 s.

Besides MC-LR, *Microcystis aeruginosa* also produces other MCs, namely MC-YR and MC-RR. Due to the similarity in the structure and molecular weight of the MC homologs, the separation of MCs is challenging [22]. The electrochemical sensors constructed with aptamers as recognition receptors produce different electrochemical responses to different homologs of MC. Therefore, the E-AB sensor constructed using the selected aptamer allows for sensitive detection of MC homologs. Responses of this sensor to other MCs, including MC-RR and MC-YR, were systematically evaluated (Figure 5A). The results demonstrate that this method allows for accurate measurement of MC-LR with good anti-interference ability in the presence of other MC homologs. In addition, the results prove the specificity of the aptamer toward MC-LR as no recognition response to other MC homologs was recorded.

The stability of the sensor was also evaluated, which revealed that the current response decreased by only 1% (Figure 5B) after storing the E-AB electrode in a Tris-HCl buffered solution at 4 °C for up to four weeks, demonstrating good stability.

The reusability of a renewable electrochemical biosensor was evaluated to verify the potential regeneration and reuse of the sensors, which can lower detection costs. The sensor is regenerable and may be used up to five times, as depicted in Figure 5C. Additionally, a minor deterioration of the monolayer and inadequate removal of the target following numerous flushing cycles could result in minor alterations in sensor performance [34].

### 3.6. Real Sample Detection in Simulated Water Samples

We examined the response of the designed sensor to MC-LR in a simulated pond water sample (pH 7.5, filtered using a filter membrane) and tap water to assess the practicality of the sensor for actual applications. Using the standard addition method, MC-LR with known concentration was spiked into tap water and pond water samples for simulations of water samples in the laboratory. The availability of the sensor was evaluated by measuring different concentrations of MC-LR in the simulation environment. The results from detecting MC-LR in simulated water samples using the sensor are presented in Table 2. We demonstrate that this sensor can detect MC-LR in two simulated complex samples using the titration curve obtained in Figure 4.

## 4. Conclusions

In summary, we developed a specific platform to detect MC-LR using an innovative combination of an MB-modified aptamer and a gold electrode. The signal generation (signal enhancement) of these sensors is associated with specific, binding-induced changes in the position of the attached MB tag, which facilitates the ET between the MB and the electrode surface. Adding MC-LR leads to a significant increase in MB current signal, thus allowing the accurate and sensitive detection of MC-LR in SWV analysis. The developed E-AB sensor exhibits a fast response time and can be regenerated at least five times with minimum signs of sensor degradation. With further optimization, this rapid, reagent-free, and sensitive electrochemical sensing platform will facilitate routine monitoring of MC-LR in a complex water environment.

## Figures and Tables

**Table 1:** Comparison in performance of electrochemical biosensors for detection of MC−LR.

Electrode	Signal Labels	Linear Range	Detection Range (LOD)	Recovery (%)	Detection Techniques	Ref
Gold-GNPs	mAb	0.05–15 µg/L	20 ng/L	95.6−105	DPV	[9]
SWCNT/SiO_2_/Go ld	mAb	1−1000 ng/L	0.6 ng/L	84.7–124.2	Electrical resistance	[14]
AuNPs/GCE	HRP-mAb	0.01−100 µg/L	4 ng/L	94.1−98.1	EIS	[32]
Au (Non-labeled)	Ab, Au NPs	0.05−15.00 μg/L	20 ng/L	95.6−105	DPV	[9]
AuNp-polyDPB- G-AuNP/GCE	Polyclonal Ab	0.1−8 pg/L	0.037 pg/L	96.3–105.8	DPV	[16]
GSs-CS/GCE	HRP-CNS-Ab	0.05−15 µg/L	16 ng/L	88–107.8	DPV	[17]
CNT@Co silicate	Multi-HRP- (Fe_3_O_4_@PDA-Au)- Ab	0.005−50 µg/L	4 ng/L	91.6–110.7	CV	[18]
GS/GCE	Ab_1_, PtRu-Ab_2_	0.01−28 ng/mL	9.63 pg/mL	99.5–102	Chronoamper ometry	[8]
CNFs/PEG/GCE	Ab_1_, Au-Ab_2_	0.0025−5 µg/L	1.68 ng/L	98−99.2	DPV	[15]
GCE modified	aptamer	0.1−1.1 µg/L	40 pg/mL	94.3−115	CV	[21]
GSPE	Ferrocene, aptamer	0.1−1000 ng/L	1.9 ng/L	91.7	SWV	[4]
Au (Non-labeled)	aptamer	1.0 × 10^−7^−5.0 × 10^−11^ mol/L	1.8 × 10^−11^ mol/L	91.2−113.7	EIS	[33]
Au (Labeled)	aptamer	0.001−0.75 ng/L	0.53 pg/mL	96.11−108.43	SWV	this method

**Table 2:** The recoveries of simulated samples were detected by labeled E−AB sensor.

Sample	[MC-LR] Added (ng/L)	[MC-LR] Detected (ng/L)	Recovery %	RDS %
Tap water	20	19.18	96.11	1.3
	150	147.75	102.36	2.7
	500	494.45	98.89	3.3
The pond water	5	5.14	102.87	1.03
	20	21.12	105.60	1.56
	150	150.49	100.33	0.33
	200	197.33	98.66	0.99
	500	497.19	99.44	1.65

## Supplementary Materials

The following supporting information can be downloaded at: https://www.mdpi.com/article/10.3390/bios12080556/s1, Figure S1: CVs of the bare Au sensor in 20 mM Tris-HCl buffer; Figure S2: CVs of the E-AB sensor in 20 mM Tris-HCl buffer; (B) SWVs of the E-AB sensor in Tris-HCl buffer (black line) and presence of MC-LR (red line); Figure S3: The potential moves with the increase of pH value; Figure S4: SWVs of the E-AB sensor in Tris-HCl buffer (black line) and presence of MC-LR (red line) with (A) 10 Hz; (B) 30 Hz; (C) 60 Hz; (D) 90 Hz; and (E) 120 Hz measurement frequency. All condition optimization is carried out with 100 ng/L MC-LR.
