# Supplementary material for: Sensitive Identification of Microcystin-LR via a Reagent-Free and Reusable Electrochemical Biosensor Using a Methylene Blue-Labeled Aptamer
Source: Biosensors (Basel). 2022 Jul 22;12(8):556. doi: 10.3390/bios12080556 (PMC9332554; doi:10.3390/bios12080556)
Supplement: Supplementary file 1 [file biosensors-12-00556-s001.zip › biosensors-1740198-supplementary.pdf]

SI APPENDIX

Sensitive Identification of Microcystin-LR via a Reagent-Free and Reusable Electrochemical Biosensor Using a Methylene Blue-Labeled Aptamer

Xiaoqian Wei [1,2,†], Shanlin Wang [1,2,†], Yujuan Zhan [1,2], Tianhan Kai [1,2,*] and Ping Ding [1,2,*]

1 Xiang Ya School of Public Health, Central South University, Changsha 410078, China; xiaoqianwei@csu.edu.cn (X.W.); 18273149872@163.com (S.W.); 216911040@csu.edu.cn (Y.Z.);
2 Hunan Provincial Key Laboratory of Clinical Epidemiology, Changsha 410078, China
† These authors contributed equally to this work.
* Correspondence: pingshui@csu.edu.cn (P.D.); th_kai@csu.edu.cn (T.K.)

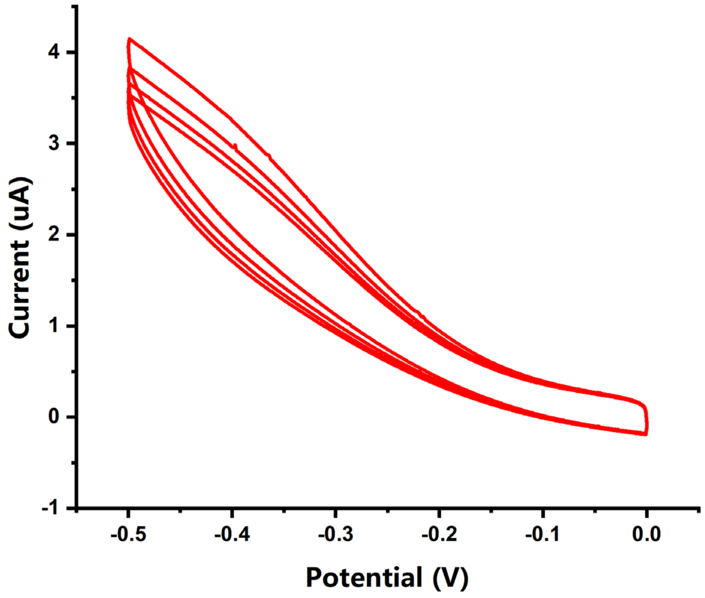

Figure S1. CVs of the bare Au sensor in 20 mM Tris-HCl buffer

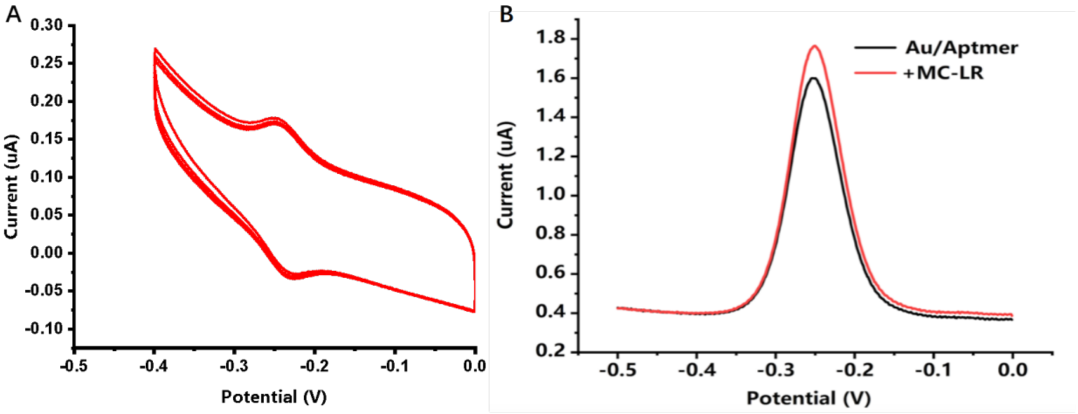

Figure S2. (A) CVs of the E-AB sensor in 20 mM Tris-HCl buffer; **(B) SWVs** of the E-AB sensor in Tris-HCl buffer (black line) and presence of MC-LR (red line).

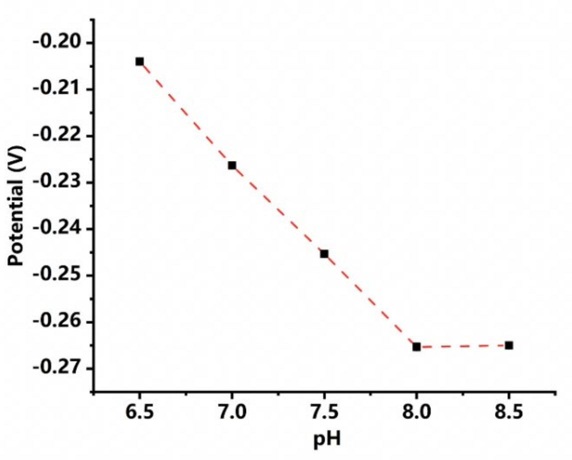

Figure S3. The potential moves with the increase of pH value.

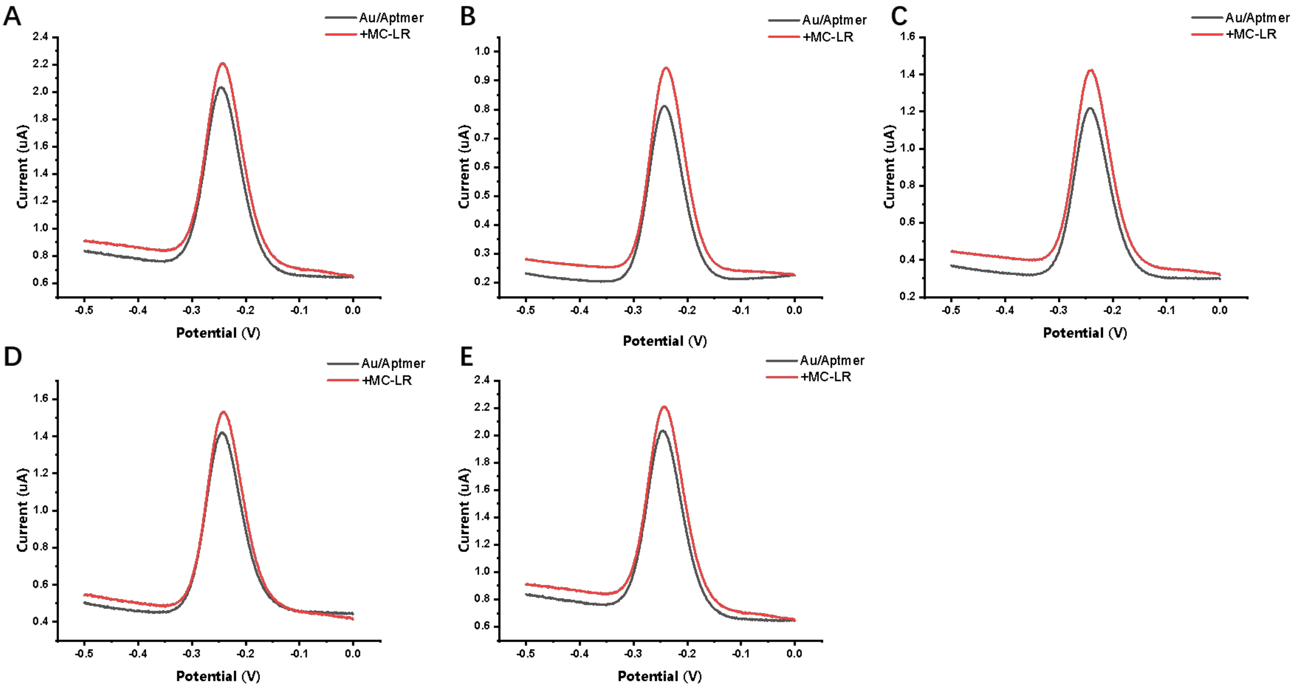

Figure S4. SWVs of the E-AB sensor in Tris-HCl buffer (black line) and presence of MC-LR (red line) with **(A)** 10 Hz; **(B)** 30 Hz; **(C)** 60 Hz; **(D)** 90 Hz; and **(E)** 120 Hz measurement frequency. All condition optimization is carried out with 100 ng/L MC-LR.
